# Socioeconomic status is inversely associated with esophageal squamous cell carcinoma risk: results from a population-based case-control study in China

**DOI:** 10.18632/oncotarget.24003

**Published:** 2018-01-06

**Authors:** Peipei Gao, Xiaorong Yang, Chen Suo, Ziyu Yuan, Hongwei Cheng, Yuechan Zhang, Li Jin, Ming Lu, Xingdong Chen, Weimin Ye

**Affiliations:** ^1^ The State Key Laboratory of Genetic Engineering, Collaborative Innovation Center for Genetics and Development, School of Life Sciences, Fudan University, Shanghai, China; ^2^ Department of Epidemiology, Shandong University, Jinan, China; ^3^ Fudan University Taizhou Institute of Health Sciences, Taizhou, China; ^4^ Taixing People's Hospital, Taixing, China; ^5^ Clinical Epidemiology Unit, Qilu Hospital of Shandong University, Jinan, China; ^6^ Department of Medical Epidemiology and Biostatistics, Karolinska Institutet, Stockholm, Sweden

**Keywords:** socioeconomic status, esophageal squamous cell carcinoma, case-control study, multiple correspondence analysis, wealth score

## Abstract

Socioeconomic status (SES) is suspected to influence the risk of esophageal squamous-cell carcinoma (ESCC) in China, however, the evidence is still inconclusive and the selection of SES indicators remains inconsistent. In current study, we examined the association between SES and risk of ESCC based on a population-based case-control study in Taixing, China, with 1298 histopathology-confirmed cases and 1900 controls recruited between October 2010 and September 2013. Data on SES indicators was collected using a structured questionnaire. We constructed a composite wealth score based on the ownership of a series of household appliances and other variables by using multiple correspondence analysis (MCA). We used unconditional logistic regression to estimate odds ratios (ORs) with 95% confidence intervals (CIs) of ESCC in association with SES indicators. SES was inversely associated with ESCC risk in current study. Higher education (secondary high school or above vs illiteracy, OR=0.60, 95%CI, 0.41-0.87), larger house area per person (>70 vs <45 square meters, OR=0.71, 95%CI, 0.59-0.86) and higher wealth score (5^th^ quintile (high) vs 1^st^ quintile (low), OR=0.43, 95%CI, 0.32-0.57) were associated with a decreased risk of ESCC. Subjects possessing several household appliances >5 years also had a lower ESCC risk. Whereas physical labor (very active vs sedentary, OR=1.69, 95%CI, 1.27-2.26) and larger families (≥6 vs <3 in household, OR=1.63, 95%CI, 1.30-2.03) increased the risk of ESCC. These findings confirm the strong inverse association between SES and ESCC risk. Future studies are needed to verify these findings and identify contributing factors underlying the observed associations.

## INTRODUCTION

Esophageal cancer is the eighth most common cancer around the world, with an estimated 456 000 new cases in 2012 [1]. There are two main histopathological types of esophageal cancer, i.e. esophageal squamous cell carcinoma (ESCC) and esophageal adenocarcinoma (EAC), each with distinct etiologies and specific risk factors [2]. About 80% of the global ESCC cases occur in the Central and South-East Asian region, and China alone contributes over half of the global ESCC cases [3]. In the past decades, the incidence of ESCC tended to decrease in developing countries such as China [4] and Iran [5], as well as developed countries such as the US [6]. Improved socioeconomic status (SES) may be one of the factors responsible for the decreasing trend of ESCC observed in developing countries [7].

An inverse association between SES and ESCC risk has been reported from Iran [8], India [9] and Sweden [10]. However, previous studies about SES and ESCC conducted in China showed inconsistent results. For example, education or income was found to be inversely associated with ESCC risk in Linzhou [11] and Heilongjiang [12], while such association was not observed in Shanxi [13]. The selection of SES indicators may matter. SES is an aggregate concept taking into account two main areas: material and social assets (material goods and social relationships), and knowledge (education and income) [14]. Traditional SES measures include education, occupation and income [15]. However, education and income may not reflect the true economic status in certain populations, especially those unemployed elders. Moreover, personal income is a sensitive topic that people may be reluctant to cooperate to report in epidemiological studies [16]. Therefore, defining proper indicators of SES is crucial in epidemiological studies for the association of SES and ESCC risk.

Secular trend analysis showed that the incidence rates of ESCC decreased since the late 1990s in China, and improved nutrition and adoption of healthy lifestyle due to the better economy, has been proposed to explain this [17, 18]. However, systematic studies for this hypothesis are lacking. Taixing in Jiangsu province, eastern China, is a high incidence area of ESCC. Since 2009, we have carried out a population-based case-control study on the etiology of upper gastrointestinal tract cancer in this high-incidence area, with a stringent study design and a relatively large sample size. Based on the collected questionnaire data during the phase I study (between September 2009 and August 2011), we have reported preliminarily an inverse association between SES and ESCC risk [19]. We now have extended the study for another 1.5 years, and based on the expanded materials, we applied a novel method to measure SES [20] and systematically examine the association between SES and ESCC risk.

## RESULTS

This study was based on 1298 ESCC cases and 1900 controls. The distribution of demographic variables of cases and controls is shown in Table 1. For both cases and controls, the mean age was 66 years, about 30% women, and more than 95% of cases and controls from rural areas. Smokers, alcohol drinkers, and tea drinkers were more prevalent in cases than in controls. Whereas the body mass index of 10 years ago and daily frequency of brushing teeth were all higher in controls than in cases.

**Table 1:** Characteristics of 1298 esophageal squamous cell carcinoma (ESCC) cases and 1900 controls in Taixing, China, 2010-2013

Characteristics	Cases (%) (n=1298)	Controls (%) (n=1900)	*P*-value
**Age (mean ± SD, years)**	66.41±8.38	66.24±8.77	0.966
**Sex**			
Men	889(68.5)	1319(69.4)	0.603
Women	409(31.5)	581(30.6)	
**Place of residence**			
Urban	52(4.0)	65(3.4)	0.454
Rural	1243(95.8)	1823(95.9)	
Missing	3(0.2)	12(0.6)	
**Marital status**			
Unmarried	55(4.2)	64(3.4)	0.117
Married	1001(77.1)	1521(80.1)	
Divorced/Widowed	242(18.6)	315(16.6)	
**Family history of esophageal cancer among first-degree relatives**			
No	874(67.3)	1542(81.2)	<0.001^**^
Yes	410(31.6)	350(18.4)	
Missing	14(1.1)	8(0.4)	
**Smoking (pack-years)**			
Never	529(40.8)	844(44.4)	<0.001^**^
≤30.0	305(23.5)	521(27.4)	
>30.0	435(33.5)	519(27.3)	
Missing	29(2.2)	16(0.8)	
**Alcohol drinking (g/d)**			
Never	596(45.9)	1107(58.3)	<0.001^**^
≤82.5	414(31.9)	501(26.4)	
>82.5	262(20.2)	275(14.5)	
Missing	26(2.0)	17(0.9)	
**Tea drinking**			
No	854(65.8)	1371(72.2)	<0.001^**^
Yes	419(32.3)	513(27.0)	
Missing	25(1.9)	16(0.8)	
**Body mass index of 10 years ago**			
<18.5	122(9.4)	110(5.8)	<0.001^**^
18.5-23.9	836(64.4)	1150(60.5)	
≥24	340(26.2)	640(33.7)	
**Sum of missing and filled teeth**			
None	287(22.1)	479(25.2)	0.012^*^
<6	436(33.6)	688(36.2)	
≥6	555(42.8)	722(38.0)	
Missing	20(1.5)	11(0.6)	
**Daily frequency of brushing teeth**			
<2	1042(80.3)	1242(65.4)	<0.001^**^
≥2	246(19.0)	648(34.1)	
Missing	10(0.8)	10(0.5)	

SD: standard deviation.

^*^P values < 0.05.

^**^P values < 0.01.

**Figure 1:**
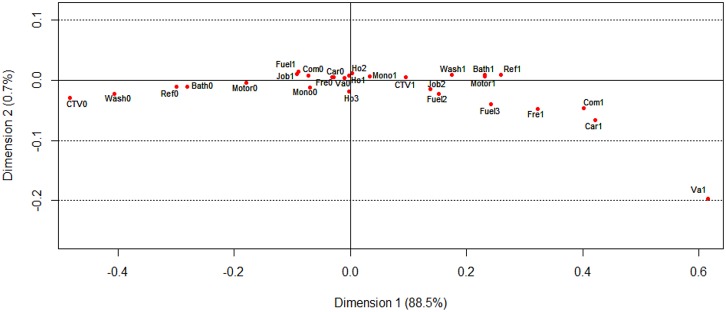
Visualization of the coordinates of wealth score-related variables in the MCA of 1900 controls, Taixing, China, 2010-2013 Appliance ownership: ‘0’ means not owning or owning the corresponding household appliance ≤ 5 years, ‘1’ means owning the corresponding household appliance > 5 years; Ca = car in the residence; Motor = motorbike; Mono = monochromatic television; CTV = color television; Wash = washing machine; Bath = bathroom; Va = vacuum; Ref = refrigerator; Fre = freezer; Com = computer. House area per person: Ho1 = house area per person < 45 square meters; Ho2 = house area per person between 45 and 70 square meters; Ho3 = house area per person > 70 square meters. Job type: Job1 = farmer; Job2 = non-farmer. Cook fuel: Fuel1 = wood; Fuel2 = coal; Fuel3 = gas or electricity.

The results of the first two dimensions of multiple correspondence analysis (MCA) among controls are shown in Figure 1. Indicators of higher wealth are located on the right side of the graph. For example, owning a vacuum for more than five years is on the rightmost side of the graph, meaning that vacuum was owned by relatively rich residents for more than 5 years. On the contrary, poor people always did not own or owned a car ≤ 5 years in the residence.

Table 2 presents odds ratios (ORs) with 95% confidence intervals (CIs) for ESCC risk in association with multiple SES indicators. Education was inversely associated with ESCC risk. In the fully-adjusted model, ORs of education were 0.74 (95%CI, 0.60-0.92) for primary school, 0.60 (95%CI, 0.46-0.78) for primary high school, and 0.60 (95%CI, 0.41-0.87) for secondary high school or above, compared with illiteracy (*P*-trend<0.001). Over 60% of study subjects were farmers, and those non-farmers showed a lower risk of ESCC. Compared with sedentary jobs, those very active in physical labor had a 69% excess risk of ESCC (OR=1.69, 95%CI, 1.27-2.26). An increased risk was also observed in those with larger families (OR=1.63, 95%CI, 1.30-2.03), while no significant association was observed for the number of siblings and children. House area above 70 square meters per person (OR=0.71, 95%CI, 0.59-0.86) and using gas or electricity as cooking fuel (OR=0.65, 95%CI, 0.53-0.81) were both associated with decreased risks of ESCC. The number of years using tap water showed no significant effects. Owning certain household appliances, including private car, motor, washing machine, refrigerator, freezer, computer, and bathroom, for more than five years in the residence was inversely associated with ESCC risk. Similarly, subjects with a higher wealth score also had a decreased risk of ESCC. Compared with the lowest quintile of total wealth score, ORs dropped from 1.00 (95%CI, 0.81-1.27) to 0.43 (95%CI, 0.32-0.57) for the 2^nd^ and the 5^th^ quintile (*P*-trend<0.001).

**Table 2:** Association between indicators of socioeconomic status (SES) and esophageal squamous cell carcinoma (ESCC) risk in Taixing, China, 2010-2013

Characteristics	Cases (%) (n=1298)	Controls (%) (n=1900)	Minimally-adjusted OR (95%CI)^a^	Fully-adjusted (except education and total wealth score) OR (95%CI)^b^	Fully-adjusted OR (95%CI)^c^
**Education**					
Illiteracy	451 (34.7)	513 (27.0)	1.00	1.00	1.00
Primary school	484 (37.3)	724 (38.1)	**0.66 (0.54-0.80)**	**0.71 (0.57-0.88)**	**0.74 (0.60-0.92)**
Primary high school	282 (21.7)	507 (26.7)	**0.52 (0.41-0.66)**	**0.55 (0.42-0.71)**	**0.60 (0.46-0.78)**
Secondary high school or above	81 (6.2)	156 (8.2)	0.48 (0.35-0.67)	0.53 (0.37-0.76)	0.60 (0.41-0.87)
*P* for trend			<0.001^**^	<0.001^**^	<0.001^**^
**Education of head of household**					
Illiteracy	250 (19.3)	234 (12.3)	1.00	1.00	1.00
Primary school	482 (37.1)	680 (35.8)	**0.66 (0.53-0.82)**	**0.68 (0.54-0.86)**	**0.71 (0.56-0.90)**
Primary high school	395 (30.4)	705 (37.1)	**0.52 (0.41-0.64)**	**0.53 (0.42-0.68)**	**0.58 (0.46-0.74)**
Secondary high school or above	171 (13.2)	281 (14.8)	**0.56 (0.43-0.73)**	**0.59 (0.44-0.78)**	**0.68 (0.51-0.92)**
*P* for trend			<0.001^**^	<0.001^**^	<0.001^**^
**Job type**					
Farmer	867 (66.8)	1135 (59.7)	1.00	1.00	1.00
Non-farmer	431 (33.2)	765 (40.3)	**0.70 (0.59-0.82)**	**0.74 (0.61-0.88)**	**0.80 (0.66-0.97)**
Worker	242 (18.6)	436 (22.9)	**0.68 (0.56-0.83)**	**0.70 (0.56-0.86)**	**0.73 (0.59-0.90)**
Service	61 (4.7)	115 (6.1)	**0.65 (0.47-0.91)**	0.69 (0.48-0.98)	0.74 (0.51-1.06)
Clerk	35 (2.7)	67 (3.5)	0.65 (0.43-0.99)	0.69 (0.44-1.09)	0.80 (0.50-1.26)
Professional	64 (4.9)	99 (5.2)	0.81 (0.58-1.13)	0.96 (0.66-1.38)	1.16 (0.79-1.70)
Administrator	29 (2.2)	48 (2.5)	0.75 (0.47-1.21)	0.88 (0.53-1.47)	1.05 (0.63-1.77)
**Physical labor**					
Sedentary	92 (7.1)	265 (14.0)	1.00	1.00	1.00
Active	113 (8.7)	283 (14.9)	1.17 (0.84-1.61)	1.09 (0.77-1.53)	0.98 (0.69-1.39)
Very active	1092 (84.2)	1347 (71.1)	**2.39 (1.86-3.09)**	**2.06 (1.56-2.71)**	**1.69 (1.27-2.26)**
*P* for trend			<0.001^**^	<0.001^**^	<0.001^**^
**Number of siblings**					
<3	393 (30.5)	648 (34.2)	1.00	1.00	1.00
3-4	545 (42.3)	794 (41.9)	1.13 (0.96-1.34)	1.10 (0.92-1.31)	1.12 (0.94-1.34)
≥5	349 (27.1)	453 (23.9)	**1.27 (1.05-1.54)**	1.15 (0.94-1.41)	1.17 (0.95-1.44)
*P* for trend			0.012^*^	0.119	0.098
**Number of children**					
<3	731 (56.8)	1065 (56.2)	1.00	1.00	1.00
3	296 (23.0)	480 (25.3)	0.88 (0.73-1.07)	0.90 (0.73-1.10)	0.91 (0.74-1.12)
≥4	260 (20.2)	350 (18.5)	1.05 (0.83-1.32)	1.09 (0.85-1.40)	1.11 (0.87-1.43)
*P* for trend			0.689	0.654	0.522
**Number of people in household**					
<3	460 (35.5)	731 (38.5)	1.00	1.00	1.00
3-5	553 (42.7)	836 (44.0)	1.06 (0.90-1.24)	1.05 (0.89-1.25)	1.16 (0.97-1.38)
≥6	281 (21.7)	331 (17.4)	**1.35 (1.11-1.65)**	**1.42 (1.15-1.76)**	**1.63 (1.30-2.03)**
*P* for trend			0.006^**^	0.003^**^	<0.001^**^
**House area (m^2^)**					
<160	514 (39.6)	648 (34.2)	1.00	1.00	1.00
160-250	463 (35.7)	646 (34.1)	0.90 (0.77-1.07)	0.91 (0.76-1.09)	0.93 (0.78-1.11)
>250	320 (24.7)	603 (31.8)	**0.67 (0.56-0.80)**	**0.69 (0.57-0.84)**	**0.72 (0.59-0.87)**
*P* for trend			<0.001^**^	<0.001^**^	0.004^**^
**House area per person (m^2^)**					
<45	492 (37.9)	599 (31.5)	1.00	1.00	1.00
45-70	447 (34.4)	664 (34.9)	**0.82 (0.69-0.97)**	**0.79 (0.66-0.95)**	**0.81 (0.68-0.97)**
>70	359 (27.7)	637 (33.5)	**0.69 (0.58-0.82)**	**0.69 (0.57-0.84)**	**0.71 (0.59-0.86)**
*P* for trend			<0.001^**^	<0.001^**^	<0.001^**^
**Use of tap water (years)**					
0	84(6.6)	85 (4.5)	1.00	1.00	1.00
1-10	723(57.1)	1042 (55.5)	**0.70 (0.51-0.96)**	0.81 (0.58-1.14)	0.91 (0.65-1.29)
>10	460(36.3)	752 (40.0)	**0.62 (0.45-0.85)**	0.74 (0.52-1.04)	0.96 (0.67-1.37)
*P* for trend			0.004^**^	0.065	0.746
**Cooking fuel**					
Wood	1023 (79.1)	1362 (71.9)	1.00	1.00	1.00
Coal	56 (4.3)	85 (4.5)	0.87 (0.62-1.24)	0.87 (0.60-1.27)	0.89 (0.61-1.31)
Gas, electricity	215 (16.6)	447 (23.6)	**0.64 (0.53-0.77)**	**0.63 (0.51-0.77)**	**0.65 (0.53-0.81)**
**Appliance ownership^d^**					
Car	29 (2.2)	123 (6.5)	**0.33 (0.22-0.50)**	**0.30 (0.20-0.46)**	**0.31 (0.20-0.48)**
Motorbike	479 (36.9)	832 (43.8)	**0.75 (0.65-0.87)**	**0.76 (0.65-0.89)**	**0.78 (0.66-0.91)**
Mono-TV	922 (71.0)	1283 (67.5)	1.19 (1.02-1.38)	1.12 (0.95-1.32)	1.14 (0.96-1.34)
CTV	1055 (81.3)	1584 (83.4)	0.87 (0.72-1.05)	0.93 (0.76-1.14)	0.98 (0.80-1.20)
Washing machine	798 (61.5)	1328 (69.9)	**0.69 (0.59-0.80)**	**0.70 (0.60-0.83)**	**0.73 (0.62-0.86)**
Vacuum	18 (1.4)	33 (1.7)	0.80 (0.45-1.44)	0.91 (0.49-1.69)	0.99 (0.53-1.85)
Refrigerator	571 (44.0)	1019 (53.6)	**0.68 (0.59-0.78)**	**0.69 (0.59-0.80)**	**0.70 (0.60-0.82)**
Freezer	78 (6.0)	166 (8.7)	**0.67 (0.51-0.89)**	**0.73 (0.54-0.98)**	**0.73 (0.53-0.99)**
Computer	95 (7.3)	293 (15.4)	**0.43 (0.34-0.55)**	**0.42 (0.32-0.55)**	**0.43 (0.33-0.57)**
Bathroom	596 (45.9)	1034 (54.4)	**0.71 (0.62-0.82)**	**0.74 (0.63-0.86)**	**0.76 (0.65-0.89)**
**Total wealth score**					
Quintile 1-lowest	312 (24.0)	355 (18.7)	1.00	1.00	1.00
Quintile 2	340 (26.0)	385 (20.3)	0.99 (0.81-1.23)	0.99 (0.80-1.26)	1.00 (0.81-1.27)
Quintile 3	287 (22.1)	405 (21.3)	**0.80 (0.64-0.98)**	0.85 (0.68-1.08)	0.88 (0.70-1.12)
Quintile 4	229 (17.6)	394 (20.7)	**0.65 (0.52-0.82)**	**0.63 (0.50-0.81)**	**0.66 (0.51-0.84)**
Quintile 5	130 (10.0)	361 (19.0)	**0.40 (0.31-0.52)**	**0.40 (0.30-0.54)**	**0.43 (0.32-0.57)**
*P* for trend			<0.001^**^	<0.001^**^	<0.001^**^

^a^Adjusted for age (continuous) and sex.

^b^Adjusted for age (continuous), sex, place of residence, marital status, family history of esophageal cancer among first-degree relatives, smoking, alcohol drinking, tea drinking, body mass index of 10 years ago, sum of missing and filled teeth, and daily frequency of brushing teeth.

^c^Besides confounders listed in model 2, further adjustment included education and total wealth score. Since total wealth score has captured information of appliance ownership, job, house area per person and cooking fuel, when analyzing these variables the the adjustment did not include total wealth score.

^d^The reference group for each appliance was the subjects who did not own or owned the appliance ≤ 5 years.

^*^P values < 0.05.

^**^P values < 0.01.

Significant ORs with 95% CIs are indicated in bold.

We examined the association between basic demographic variables, family history of esophageal cancer among first-degree relatives and lifestyle-related indicators and total wealth score (Table 3). Compared with the 1^st^ quintile, study subjects with the 5^th^ quintile of total wealth score were more likely to be younger, an alcohol drinker or tea drinker, having a higher body mass index of 10 years ago, having better oral hygiene, brushing teeth more frequently, while there was no difference in sex, family history of esophageal cancer among first-degree relatives and smoking.

**Table 3:** Association between basic demographic variables, family history of esophageal cancer among first-degree relatives and lifestyle-related indicators and total wealth score in Taixing, China, 2010-2013

Characteristics	Total wealth score					*P*-value
	Quintile 1-lowest	Quintile 2	Quintile 3	Quintile 4	Quintile 5	
**Age**						<0.001^**^
≤67	295 (16.9)	352 (20.2)	398 (22.8)	372 (21.3)	329 (18.8)	
>67	372 (25.6)	373 (25.7)	294 (20.2)	251 (17.3)	162 (11.2)	
**Sex**						0.110
Men	459 (20.8)	491 (22.2)	467 (21.2)	426 (19.3)	365 (16.5)	
Women	208 (21.0)	234 (23.6)	225 (22.7)	197 (19.9)	126 (12.7)	
**Family history of esophageal cancer among first-degree relatives**						0.088
No	487 (20.2)	552 (22.8)	523 (21.6)	467 (19.3)	387 (16.0)	
Yes	175 (23.0)	169 (22.2)	163 (21.4)	151 (19.9)	102 (13.4)	
**Smoking (pack-years)**						0.092
Never	291 (21.2)	313 (22.8)	321 (23.4)	264 (19.2)	184 (13.4)	
≤30.0	175 (21.2)	191 (23.1)	164 (19.9)	163 (19.7)	133 (16.1)	
>30.0	186 (19.5)	210 (22.0)	203 (21.3)	187 (19.6)	168 (17.6)	
**Alcohol drinking (g/d)**						<0.001^**^
Never	363 (21.3)	396 (23.3)	384 (22.5)	323 (19.0)	237 (13.9)	
≤82.5	192 (21.0)	216 (23.6)	202 (22.1)	164 (17.9)	141 (15.4)	
>82.5	101 (18.8)	102 (19.0)	102 (19.0)	126 (23.5)	106 (19.7)	
**Tea drinking**						<0.001^**^
No	512 (23.0)	533 (24.0)	505 (22.7)	397 (17.8)	278 (12.5)	
Yes	144 (15.5)	181 (19.4)	183 (19.6)	217 (23.3)	207 (22.2)	
**Body mass index of 10 years ago**						<0.001^**^
<18.5	81 (34.9)	41 (17.7)	51 (22.0)	38 (16.4)	21 (9.1)	
18.5-23.9	428 (21.6)	468 (23.6)	439 (22.1)	360 (18.1)	291 (14.7)	
≥24	158 (16.1)	216 (22.0)	202 (20.6)	225 (23.0)	179 (18.3)	
**Sum of missing and filled teeth**						<0.001^**^
None	126 (16.4)	171 (22.3)	175 (22.8)	167 (21.8)	127 (16.6)	
<6	215 (19.1)	227 (20.2)	238 (21.2)	235 (20.9)	209 (18.6)	
≥6	321 (25.1)	321(25.1)	274 (21.5)	210 (16.4)	151 (11.8)	
**Daily frequency of brushing teeth**						<0.001^**^
<2	518 (22.7)	578 (25.3)	482 (21.1)	423 (18.5)	283 (12.4)	
≥2	144 (16.1)	144 (16.1)	206 (23.0)	194 (21.7)	206 (23.0)	

^**^P values < 0.01.

To further examine existence of potential effect modifiers of the association between total wealth score and ESCC, we performed stratified analyses by age, sex, family history of esophageal cancer among first-degree relatives, smoking, alcohol drinking, sum of missing and filled teeth and daily frequency of brushing teeth (Table 4). Overall, the associations between total wealth score and ESCC risk were consistent across strata (*P*-values for heterogeneity > 0.05).

**Table 4:** Association between total wealth score and esophageal squamous cell carcinoma (ESCC) risk separately stratified by basic demographic variables, family history of esophageal cancer among first-degree relatives and lifestyle-related indicators in Taixing, China, 2010-2013

Strata	Total wealth score^a^					
	Quintile 1-lowest	Quintile 2	Quintile 3	Quintile 4	Quintile 5	*P* for heterogeneity
**Age**						0.378
≤67	1.00	1.03 (0.73-1.45)	0.83 (0.59-1.16)	0.59 (0.41-0.83)	0.38 (0.26-0.56)	
>67	1.00	1.02 (0.74-1.39)	0.92 (0.66-1.29)	0.70 (0.49-1.01)	0.48 (0.30-0.75)	
**Sex**						0.987
Men	1.00	1.05 (0.79-1.39)	0.87 (0.65-1.16)	0.66 (0.48-0.89)	0.43 (0.31-0.60)	
Women	1.00	0.94 (0.63-1.40)	0.96 (0.64-1.44)	0.66 (0.43-1.02)	0.48 (0.28-0.81)	
**Family history of esophageal cancer** **among first-degree relatives**						0.140
No	1.00	0.96 (0.74-1.24)	0.94 (0.72-1.23)	0.61 (0.46-0.82)	0.42 (0.30-0.58)	
Yes	1.00	1.19 (0.74-1.92)	0.65 (0.41-1.04)	0.73 (0.45-1.19)	0.37 (0.21-0.66)	
**Smoking (pack-years)**						0.978
Never	1.00	1.06 (0.75-1.49)	0.99 (0.71-1.41)	0.73 (0.51-1.07)	0.52 (0.34-0.82)	
≤30.0	1.00	1.00 (0.63-1.58)	0.74 (0.46-1.19)	0.67 (0.41-1.09)	0.43 (0.25-0.74)	
>30.0	1.00	0.85 (0.54-1.32)	0.79 (0.50-1.24)	0.59 (0.36-0.95)	0.32 (0.19-0.55)	
**Alcohol drinking (g/day)**						0.244
Never	1.00	0.76 (0.56-1.04)	0.77 (0.56-1.05)	0.60 (0.43-0.85)	0.41 (0.27-0.62)	
≤82.5	1.00	1.51 (0.99-2.32)	0.93 (0.60-1.44)	0.86 (0.54-1.36)	0.49 (0.29-0.82)	
>82.5	1.00	1.23 (0.66-2.31)	1.22 (0.64-2.32)	0.65 (0.35-1.21)	0.46 (0.24-0.89)	
**Sum of missing and filled teeth**						0.118
None	1.00	1.63 (0.97-2.72)	1.11 (0.66-1.86)	0.87 (0.51-1.50)	0.56 (0.30-1.04)	
<6	1.00	0.67 (0.45-1.00)	0.87 (0.58-1.30)	0.51 (0.33-0.77)	0.26 (0.16-0.42)	
≥6	1.00	1.03 (0.73-1.45)	0.81 (0.56-1.16)	0.70 (0.48-1.04)	0.58 (0.37-0.92)	
**Daily frequency of brushing teeth**						0.207
<2	1.00	0.92 (0.72-1.19)	0.92 (0.71-1.20)	0.67 (0.52-0.91)	0.43 (0.31-0.60)	
≥2	1.00	1.41 (0.84-2.41)	0.80 (0.48-1.33)	0.63 (0.37-1.08)	0.41 (0.24-0.73)	

^a^Adjusted for age (continuous), sex, place of residence, marital status, family history of esophageal cancer among first-degree relatives, smoking, alcohol drinking, tea drinking, body mass index of 10 years ago, sum of missing and filled teeth, times of tooth brushing per day, daily frequency of brushing teeth, education, where appropriate.

By excluding 160 case subjects whose SES data were collected after pathological diagnosis, we performed a sensitivity analysis. The results did not change materially. For example, 9.6% of ESCC cases and 19.0% of controls were with the 5^th^ quintile of total wealth score, and the corresponding OR was 0.37 (95%CI, 0.27-0.51) compared with 1^st^ quintile after adjustment for potential confounders. Similarly, estimates for other SES indicators including education, job, family size, house condition and cooking fuel almost did not change, compared with those of the main analysis (data not shown).

## DISCUSSION

In this large population-based case-control study, we built a composite wealth score based on the ownership of a series of household appliances and other variables and confirmed a significant inverse association between SES and ESCC risk in a high-risk area for ESCC, Taixing of Jiangsu, China. Education, house area per person, use of certain cooking fuels, and especially total wealth score demonstrated a dose-response inverse association with ESCC risk. Active in physical labor and large family size were associated with an increased risk of ESCC.

Our study is a well-designed population-based case-control study on esophageal cancer. This study has several strengths. First, around 80% of the incident cases of ESCC in Taixing were recruited and the response rate of controls, which were frequency-matched and randomly selected, was relatively high, above 70%. Second, all ESCC cases were carefully confirmed by reviewing additionally collected pathological sections or original pathological reports after surgical resection by a study pathologist. Third, we used not only the ownership of household appliances but also other related variables, including education, job, family size, house condition, use of tap water and cooking fuel, as SES indicators to perform a systematic assessment of the association between SES and ESCC risk. Last, but not the least, the sample size is relatively large and we have collected detailed information about potential confounders for adjustment.

Previous studies have suggested an inverse association between SES and ESCC risk, which have been conducted in countries with different incidence rates of ESCC and economic level [8–10]. Our study results are in line with those from previous studies. The composite wealth score, based on the ownership of household appliances and other variables, was inversely associated with ESCC risk. Tobacco smoking [21], alcohol drinking [22], and poor oral health [23], which are also associated with SES, most likely affect the occurrence of ESCC, and thus the association between SES and ESCC risk may be confounded by these factors. However, the association remained statistically significant after adjustment for these and other potential confounding factors, thus SES is an independent risk factor of ESCC apart from these factors. Further studies are needed to explore other contributing factors, such as eating habits and the alteration of the distribution of oral flora or gastrointestinal flora.

Previous studies generally used education or job as SES indicators. Education contributed to a lower risk of ESCC in Sweden [24] and China [25], which were consistent with our study results. Education may reflect future occupational opportunities and potential income [15]. People with higher education are more likely to gain knowledge for promoting health [26]. And most of them have had a habit of periodic health examination and would prefer early treatment for illness. Regarding job, active physical labor was positively associated with the risk of ESCC in current study, which was similar to the study in India [9], whereas studies in the US reported none relationships [27, 28]. Jobs reflect different characteristics, rewards, qualifications and prestige [29]. In current study, active or very active physical labors were easily exposed to physical and psychosocial risks, with limited education and economy. They also always carry a higher risk of exposure to toxic material, which may be a hazard for health.

Except for education and job, we also adopt other SES indicators, including family size, house condition, use of tap water, cooking fuel, and the ownership of household appliances, which suffer less from recall bias and social desirability bias. Multiple SES indicators synthetically contribute to socioeconomic status. For example, the ownership of household appliances may act as indicators of living standards. Possessing these household appliances contributes to lower ESCC risk in a certain way. Owing private cars and motors helps relieving stress on traffic such that it makes the owners more accessible to shopping, medical attention and social activities. Refrigerators and freezers help keeping food fresh. Computers play a critical role in our daily life, which help staying updated and gaining information on health. Possessing these household appliances for a longer time allows people to get high-quality materials and better, easier and faster access to services, which are beneficial to health outcomes [16, 26].

Although SES is not a biological factor, it may have an impact on health outcome through modifying health-related behaviors and lifestyles [15]. On one hand, our results suggested that individuals with higher wealth score in Taixing tend to develop some unhealthy behaviors, such as smoking or alcohol drinking, for the need of development of social network due to local customs and culture, and these factors, particularly, alcohol drinking, are significantly associated with an increased risk of ESCC. On the other hand, higher wealth score helps local people to adopt a healthier lifestyle in other aspects, including better oral hygiene, more frequent tooth brushing and healthier eating habits, which may decrease ESCC risk. Therefore, we further examined the effect modification of the association between SES and ESCC by these unhealthy and healthy lifestyle-related factors, separately. The associations between total wealth score and ESCC risk were not statistically different between strata in the stratified analyses of sex, family history of esophageal cancer among first-degree relatives, smoking, alcohol drinking, tea drinking, body mass index of 10 years ago, sum of missing and filled teeth, and daily frequency of brushing teeth.

Our study has its limits. First, selection bias may exist in this population-based case-control study, especially in the recruitment of controls. Compared with those with higher SES or younger age, who might be more likely to refuse to cooperate due to current employment, controls with lower SES or older age were more likely to be recruited. However, this selection bias tended to drive the observed associations toward the null, resulting under-estimated ORs. Furthermore, no significant differences were observed between respondents and non-respondents for age, sex and residence. Second, recall bias is another limitation in the exposure assessment. To minimize the recall bias, we mainly selected easily collected indicators, such as education, family size and the ownership of household appliances. Most cases in this study were enrolled before they were diagnosed, such that it helps diminishing the possibility of differential recall on socioeconomic status between cases and controls. Furthermore, we obtained similar results in the sensitivity analysis by excluding case subjects enrolled after pathological diagnosis and/or treatment started, which allayed such a concern.

Although the economic level of Taixing increases fast, the incidence rate of ESCC remains relatively high, though with somewhat decreasing trend. Improved SES might contribute partially to the observed decreasing trend. Notwithstanding, the spectrum of risk factors may alter with the rapid development of the national economy; thus, originally inconspicuous factors may stand out, warranting future studies to explore.

In summary, the current study confirmed a strong inverse association between SES and ESCC risk in a high-risk area in China. Further studies are needed to explore the possible alteration of risk factors brought by economic development. This study also suggested the importance of exploring multiple SES indicators and further efforts for identifying the contributing factors underlying SES-ESCC associations are needed.

## MATERIALS AND METHODS

### Study population

We carried out this population-based case-control study on the etiology of esophageal cancer in Taixing of Jiangsu Province. Details on the study design have been reported earlier [23, 30], and the current report is based on materials collected for an extended period of 1.5 years. In brief, we gathered all suspected esophageal case subjects in endoscopy units in the four largest regional hospitals in Taixing, including the Taixing People's Hospital, the Second People's Hospital of Taixing, the Taixing Chinese Medicine Hospital and the Third People's Hospital of Taixing, between October 2010 and September 2013. To find any missing cases, we also performed cross-linkage to the local Cancer Registry. Control subjects were randomly selected every 12 months during the same period from the local Population Registry, which covers the whole population of Taixing. To increase the statistical power of the study, we employed frequency-match method for control selection (strata were defined by sex and 5-year age group). Additional eligible criteria for both cases and controls were that age between 40 and 85 and local inhabitants living in Taixing for at least 5 years.

During the 3-year period, we enrolled 1401 suspected cases by endoscopic examination. By comparing with the local Cancer Registry, we further identified 994 additional cases, among whom 291 died before being contacted, 247 were living outside the study region or too ill to participate and 176 refused, leaving 280 to be recruited into the study. We tried to collect additional pathological sections from stored formalin-fixed and paraffin-embedded tissue blocks, and collect pathological reports after surgical operation for those whose tissue blocks were unavailable. All sections stained by the H&E method, along with original pathological reports for those without available tissue blocks, were again reviewed by a pathologist. After excluding those whose diagnoses were non-cancer, or whose ages were out of the eligible range (40-85 years), we enrolled a total of 1499 esophageal cancer cases, including 1318 from the hospitals’ endoscopy units and 181 from the local Cancer Registry, into the study. According to the number of estimated total incident cases from the local Cancer Registry, about 78.3% of the incident cases in the study base were included. Among the enrolled cases, 1418 were ESCC cases and 81 non-ESCC cases.

The upper gastrointestinal tract cancer case-control study enrolled gastric cancer cases simultaneously. Since the age distribution of esophageal and gastric cancer is similar, a common set of population controls was selected. During study period, we enrolled 2699 potential esophageal and gastric cases in total, thus for each stratum (defined by sex and 5-year age group) we selected corresponding controls with a 1.3:1 ratio, considering a moderately high non-response rate among controls. To summarize, we randomly selected 3501 population-based controls, of whom 643 were excluded due to death before being contacted, outmigration or inability to be reached, leaving 2858 eligible subjects. With a participation rate of 70.4%, 2011 controls participated in this study. After excluding 19 controls out of the eligible age range (40-85 years), we recruited 1992 controls to the current study.

This analysis was based on the 1418 ESCC cases, which were independently reviewed and confirmed, and 1992 controls. After removing individuals with incomplete questionnaire information on our main exposure variables, we included 1298 ESCC cases and 1900 controls in current analysis.

This study was approved by the Institutional Review Board at the School of Life Sciences, Fudan University, and the Institutional Review Board at Qilu Hospital, Shandong University. Written informed consent was obtained from all participants.

### Data collection

Detailed information on demographic characteristics, SES indicators, and potential confounders of interest was collected using a structured electronic questionnaire in face-to-face interview.

Information about SES indicators obtained in this study includes education, job (job type and physical labor), family size (number of siblings, number of children and number of people in household), housing condition (house area and house area per person), use of tap water, cooking fuel, and ownership of several household appliances (private car, motorbike, mono-TV, CTV, washing machine, vacuum, refrigerator, freezer, computer and bathroom). Education level was classified into four categories (illiteracy, primary school, primary high school and second high school or above). Job type includes farmer and non-farmer. Non-farmer was classified into several categories based on the occupational classification in People's Republic of China [31]: worker (engaged in industrial production, transportation or equipment operation), service (work in restaurants or other commercial service units), clerk, professional (required certain professional skills), and administrator (engaged in enterprise or organization administration). Job intensity was also categorized based on physical labor: ordinary (barber, guard, engineer, programmer) which is easy for people to finish without sweating at all, active (cleaner, nurse, electrician) which needs efforts to finish with sweating a little, very active (agricultural workers, miner, builder) which is hard to finish with heart rate significantly increasing and sweating a lot. Smoker and tea drinker were defined as ever using the corresponding product respectively at least once a day for 6 months and alcohol drinker as at least once a week for 6 months.

### Statistical analysis

Distribution of demographic and SES variables by case/control status is summarized and presented as number and percentage. To test difference between cases and controls in the distribution of the variables of interest, we used Wilcoxon rank sum test for continuous variables, Pearson's Chi-squared test for categorical variables, and Kruskal-Wallis rank sum test for ordinal variables. We calculated minimally-adjusted and fully-adjusted ORs with 95% CIs by using unconditional logistic regression. Since ESCC is a rare disease (the crude incidence rate of esophageal cancer in Taixing was 60.67 per 100,000 person-years [32]), the estimated ORs can be interpreted as relative risks. Continuous variables were categorized by resulting equal numbers of controls in each category. The *p* values of trend test were obtained from Wald test in the logistic regression model by including semi-continuous variables and the *p* values of heterogeneity tests were obtained from the likelihood ratio test. We aimed to build a composite wealth score [20] for each study subject based on ownership of a series of household appliances and other variables, including job, house area per person and cooking fuel, using MCA. MCA is a generalization of correspondence analysis. It transforms a set of categorical variables to a smaller set of latent variables, which is suitable for discrete or categorical variables. It aims at providing a summarized representation of a high number of variables. The main output statistics of MCA are eigenvalues and inertia. Inertia is a scaling of the Pearson's chi-squared statistic, which shows a linear combination of elements that measure the distance of each contingency cell entry from independence. MCA provides a geometric representation of data structures. The graph indicates the relationship between variables, represented by dots, in a two-dimensional or three-dimensional space. In current study, we selected the parameters produced by 1900 controls as standard references, for the need of standard eigenvalues and inertia to calculate a composite wealth score. In addition, we used the parameters in the first dimension to construct the total wealth score, for which the first dimension captures the vast majority (88.5%) of inertia (variance) of the characteristics in ownership of the household appliances and other variables [33].

In the multivariate analysis, we adjusted for age (continuous), sex (men/women), place of residence (urban/rural), marital status (unmarried/married/divorced or widowed), family history of esophageal cancer among first-degree relatives (yes/no), smoking (never/≤30.0 pack-years/>30.0 pack-years), alcohol drinking (never/≤82.5 g/d/>82.5 g/d), tea drinking (never/ever), body mass index of 10 years ago (<18.5/18.5-23.9/≥24), sum of missing and filled teeth (none/<6/≥6), and daily frequency of brushing teeth (<2/≥2). Several indicators are correlated with socioeconomic status, including family size, housing condition, use of tap water, cooking fuel and household appliances ownership, thus not adjusted. Analyses were performed using R statistical software (version 3.3.2). Two-sided *p* values < 0.05 were considered statistically significant.

## Abbreviations

SES: socioeconomic status
ESCC: esophageal squamous cell carcinoma
MCA: multiple correspondence analysis
OR: odds ratio
CI: confidence interval
SD: standard deviation

## References

[1] Ferlay J, Soerjomataram I, Dikshit R, Eser S, Mathers C, Rebelo M, Parkin DM, Forman D, Bray F (2015). Cancer incidence and mortality worldwide: sources, methods and major patterns in GLOBOCAN 2012. Int J Cancer.

[2] Enzinger PC, Mayer RJ (2003). Esophageal cancer. N Engl J Med.

[3] Arnold M, Soerjomataram I, Ferlay J, Forman D (2015). Global incidence of oesophageal cancer by histological subtype in 2012. Gut.

[4] Wei WQ, Yang J, Zhang SW, Chen WQ, Qiao YL (2011). Esophageal cancer mortality trends during the last 30 years in high risk areas in china: comparison of results from national death surveys conducted in the 1970’s, 1990’s and 2004-2005. Asian Pac J Cancer Prev.

[5] Semnani S, Sadjadi A, Fahimi S, Nouraie M, Naeimi M, Kabir J, Fakheri H, Saadatnia H, Ghavamnasiri MR, Malekzadeh R (2006). Declining incidence of esophageal cancer in the turkmen plain, eastern part of the caspian littoral of iran: a retrospective cancer surveillance. Cancer Detect Prev.

[6] Trivers KF, Sabatino SA, Stewart SL (2008). Trends in esophageal cancer incidence by histology, United States, 1998-2003. Int J Cancer.

[7] Ng CJ, Teo CH, Abdullah N, Tan WP, Tan HM (2015). Relationships between cancer pattern, country income and geographical region in Asia. BMC Cancer.

[8] Islami F, Kamangar F, Nasrollahzadeh D, Aghcheli K, Sotoudeh M, Abedi-Ardekani B, Merat S, Nasseri-Moghaddam S, Semnani S, Sepehr A, Wakefield J, Moller H, Abnet CC (2009). Socio-economic status and oesophageal cancer: results from a population-based case-control study in a high-risk area. Int J Epidemiol.

[9] Dar NA, Shah IA, Bhat GA, Makhdoomi MA, Iqbal B, Rafiq R, Nisar I, Bhat AB, Nabi S, Masood A, Shah SA, Lone MM, Zargar SA (2013). Socioeconomic status and esophageal squamous cell carcinoma risk in kashmir, india. Cancer Sci.

[10] Lagergren J, Andersson G, Talback M, Drefahl S, Bihagen E, Harkonen J, Feychting M, Ljung R (2016). Marital status, education, and income in relation to the risk of esophageal and gastric cancer by histological type and site. Cancer.

[11] Tran GD, Sun XD, Abnet CC, Fan JH, Dawsey SM, Dong ZW, Mark SD, Qiao YL, Taylor PR (2005). Prospective study of risk factors for esophageal and gastric cancers in the linxian general population trial cohort in china. Int J Cancer.

[12] Hu J, Nyren O, Wolk A, Bergstrom R, Yuen J, Adami HO, Guo L, Li H, Huang G, Xu X, Zhao F, Chen Y, Wang C (1994). Risk factors for oesophageal cancer in northeast china. Int J Cancer.

[13] Gao Y, Hu N, Han XY, Ding T, Giffen C, Goldstein AM, Taylor PR (2011). Risk factors for esophageal and gastric cancers in shanxi province, china: a case-control study. Cancer Epidemiol.

[14] Krieger N, Williams DR, Moss NE (1997). Measuring social class in US public health research: concepts, methodologies, and guidelines. Annu Rev Public Health.

[15] Adler NE, Newman K (2002). Socioeconomic disparities in health: pathways and policies. Health Affairs.

[16] Galobardes B, Lynch J, Smith GD (2007). Measuring socioeconomic position in health research. Br Med Bull.

[17] Guo P, Li K (2012). Trends in esophageal cancer mortality in china during 1987-2009: age, period and birth cohort analyzes. Cancer Epidemiol.

[18] Zhao J, He YT, Zheng RS, Zhang SW, Chen WQ (2012). Analysis of esophageal cancer time trends in china, 1989-2008. Asian Pac J Cancer Prev.

[19] Zhang L, Cheng H, Zhou Y, Yuan Z, Chen T, Chen X, Lu M (2014). Association between socioeconomic status and esophageal squamous cell carcinoma in the population of taixing area, jiangsu procince. Chin J Epidemiol.

[20] Rutstein SO, Johnson K (2004). The DHS wealth index. DHS Comparative Reports No. 6. Calverton.

[21] Okello S, Churchill C, Owori R, Nasasira B, Tumuhimbise C, Abonga CL, Mutiibwa D, Christiani DC, Corey KE (2016). Population attributable fraction of esophageal squamous cell carcinoma due to smoking and alcohol in Uganda. BMC Cancer.

[22] Vioque J, Barber X, Bolumar F, Porta M, Santibanez M, de la Hera MG, Moreno-Osset E (2008). Esophageal cancer risk by type of alcohol drinking and smoking: a case-control study in spain. BMC Cancer.

[23] Chen X, Yuan Z, Lu M, Zhang Y, Jin L, Ye W (2017). Poor oral health is associated with an increased risk of esophageal squamous cell carcinoma - a population-based case-control study in china. Int J Cancer.

[24] Ljung R, Drefahl S, Andersson G, Lagergren J (2013). Socio-demographic and geographical factors in esophageal and gastric cancer mortality in sweden. PLoS One.

[25] Miao J, Ming S, Zhong JX, Xu H, Ming W, Yi ZJ, Qiang HR, Kang WS, Ju SG (2013). A case-control study on risk factors for esophageal cancer in huai’an district of jiangsu province, china. Tumor.

[26] Braveman PA, Cubbin C, Egerter S, Chideya S, Marchi KS, Metzler M, Posner S (2005). Socioeconomic status in health research: one size does not fit all. Jama.

[27] Leitzmann MF, Koebnick C, Freedman ND, Park Y, Ballard-Barbash R, Hollenbeck A, Schatzkin A, Abnet CC (2009). Physical activity and esophageal and gastric carcinoma in a large prospective study. Am J Prev Med.

[28] Cook MB, Matthews CE, Gunja MZ, Abid Z, Freedman ND, Abnet CC (2013). Physical activity and sedentary behavior in relation to esophageal and gastric cancers in the NIH-AARP cohort. PLoS One.

[29] Gregorio DI, Walsh SJ, Paturzo D (1997). The effects of occupation-based social position on mortality in a large american cohort. Am J Public Health.

[30] Chen T, Cheng H, Chen X, Yuan Z, Yang X, Zhuang M, Lu M, Jin L, Ye W (2015). Family history of esophageal cancer increases the risk of esophageal squamous cell carcinoma. Sci Rep.

[31] PRC Grand Classification of Occupations (2015). ed. Beijing: china human resources and social security publishing group co. Ltd.

[32] Chen J, Zhou Y, Liu H, Chen T, Yuan Z, Jiang Y, Chen X, Lu M (2014). [Trend analysis of esophageal cancer incidence in taixing city, jiangsu province of china from 2003 to 2010]. [Article in Chinese]. Fudan Univ J Med Sci.

[33] Booysen F, van der Berg S, Burger R, von Maltitz M, du Rand G (2008). Using an asset index to assess trends in poverty in seven sub-saharan african countries. World Dev.

